# Characteristics of Children Hospitalized for Pandemic (H1N1) 2009, Malaysia

**DOI:** 10.3201/eid1704.101212

**Published:** 2011-04

**Authors:** Hussain Imam Muhammad Ismail, Kah Kee Tan, Yin Leng Lee, Wilson S.C. Pau, Kamarul A.M. Razali, Thahira Mohamed, Tassha Adnan, Premaa Subramaniam, Jamaiyah Hanif

**Affiliations:** Author affiliations: Hospital Kuala Lumpur, Kuala Lumpur, Malaysia (H.I.M. Ismail, K.A.M. Razali, T. Mohamed);; Hospital Tuanku Ja’afar, Seremban, Malaysia (K.K. Tan, W.S.C. Pau);; Ministry of Health Malaysia, Kuala Lumpur (Y.L. Lee, T. Adnan, P. Subramaniam, J. Hanif)

**Keywords:** Children, Malaysia, tropical, hospitalized, co-morbidities, death, influenza, viruses, pandemic (H1N1) 2009, dispatch

## Abstract

To determine effects of pandemic (H1N1) 2009 on children in the tropics, we examined characteristics of children hospitalized for this disease in Malaysia. Of 1,362 children, 51 (3.7%) died, 46 of whom were in an intensive care unit. Although disease was usually mild, >1 concurrent conditions were associated with higher death rates.

Transmission of pandemic (H1N1) 2009 in the Northern and Southern Hemispheres has been well documented (*1**–**12*). These reports described a relatively mild and self-limited clinical illness for most cases. However, data on disease prevalence and severity in children in the tropics are scarce. Malaysia has 132 public and 214 private hospitals. We examined the demographics, clinical presentation, and outcomes of children hospitalized for pandemic (H1N1) 2009 in 68 Ministry of Health–affiliated public hospitals in Malaysia that offered pediatric services. These 68 hospitals provide 3,757 beds for children and 101 beds in pediatric intensive care units; each year they serve ≈7,898,700 children <12 years of age. During the pandemic (H1N1) 2009 containment phase, May–July 2009, the Ministry of Health mandated that all patients with influenza-like illness (ILI) be admitted to government (public) hospitals for observation and management. During the mitigation phase from August 2009 on, only patients with moderate to severe cases of ILI were hospitalized (*13*). Influenza vaccine is used in the private sector only, and during the study period, only health care workers were vaccinated against pandemic (H1N1) 2009.

## The Study

We enrolled children <12 years of age who were hospitalized for ILI from June 18, 2009, through March 1, 2010, and for whom pandemic (H1N1) 2009 infection was confirmed by real-time reverse transcription–PCR. This study was reviewed and approved by the Malaysian Research and Ethics Committee. Informed consent was provided for patients with confirmed diagnoses.

During the study period, 1,362 children were hospitalized for ILI. The first case was diagnosed and confirmed as pandemic (H1N1) 2009 during the third week of June 2009; the number of cases peaked at week 33 and declined until week 43 of 2009 and weeks 1–9 of 2010 (Figure). The rapid decline of cases after week 33 may have resulted from a change in hospitalization criteria recommendations. From June 18 through July 2009, hospitalization rates among children <12 years, <5 years, and <2 years of age were 1.4, 1.0, and 1.1 per 100,000 children in each age group, respectively. From August 2009 through February 2010, corresponding hospitalization rates were 15.9, 23.8, and 33 per 100,000 children, respectively.

**Figure:**
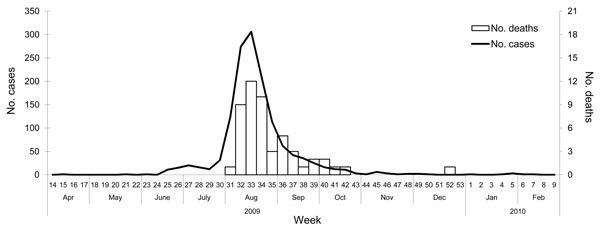
Distribution of laboratory-confirmed cases of pandemic (H1N1) 2009 and deaths in 1,362 hospitalized children, Malaysia, June 18, 2009–March 1, 2010.

Overall median age of the hospitalized children was 3 years (interquartile range [IQR] 1–6 years); 861 (63.2%) were <5 years and 536 (39.4%) were <2 years of age. Among those who died, median age at time of death was 2 years (IQR 0–6 years). Other demographic characteristics of the cohort are shown in Table 1.

**Table 1:** Characteristics of 1,362 hospitalized children with pandemic (H1N1) 2009, Malaysia, June 18, 2009–March 1, 2010

Characteristic	No. (%) children
Demographic	
Male sex	762 (55.9)
Age group	
0–6 mo	152 (11.2)
7–12 mo	182 (13.4)
13–23 mo	202 (14.8)
2–4 y	325 (23.9)
5–8 y	298 (21.9)
9–12 y	203 (14.8)
Ethnic group	
Malay	995 (73.1)
Chinese	109 (8.0)
Indian	83 (6.1)
Native East Malaysian*	99 (7.3)
Indigenous native	24 (1.8)
Other	52 (3.8)
Clinical sign or symptom	
Fever	1313 (96.4)
Cough	1237 (90.8)
Runny nose	794 (58.3)
Nausea	346 (25.4)
Poor feeding	310 (22.8)
Labored breathing	293 (21.5)
Diarrhea	177 (13.0)
Sore throat	164 (12.0)
Seizure	117 (8.6)
Fatigue	94 (6.9)
Headache	30 (2.2)
Abdominal pain	30 (2.2)
Altered consciousness	13 (1.0)
Vomiting	6 (0.4)
Disease severity/treatment needed	
Admission to intensive care unit	134 (9.8)
Mechanical ventilation	101 (7.4)
Supplemental oxygen†	317 (23.3)
Noninvasive ventilation‡	4 (0.3)
Complication	
Shock	57 (4.2)
Acute respiratory distress syndrome	41 (3.0)
Encephalitis/encephalopathy§	21 (1.5)
Myocarditis	8 (0.6)
Disseminated intravascular coagulation	7 (0.5)
Liver impairment	32 (2.3)
Multiple organ failure	12 (0.9)
Myoglobinuria	1 (0.07)

*Kadazan/Dusun, Melanau, Bajau, Bidayuh, Iban, Orang Ulu, Lundayeh, Kayan, Kedayan, Sabahan, Kadayan, Suluk, Tidung, Bisaya. †Oxygen delivered by nasal cannula, nasal prong, or face mask. ‡Mechanical ventilation that does not use an artificial airway such as endotracheal tube. §Inflammation of brain or degeneration of brain function.

A total of 602 (44.2%) children were admitted to hospital within 48 hours of onset of clinical signs. Median interval from onset of signs to hospitalization was 3 days (IQR 1–5 days) for the overall cohort, 3 days (IQR 1–5 days) for those who survived, and 4 days (IQR 2–6 days) for those who died. Among 120 (8.8%) children whose clinical condition worsened during hospitalization, deterioration occurred within the first 24 hours after admission for 67 (55.9%). Among 657 (48.2%) patients for whom blood cultures were performed, results were positive for only 29 (4.4%). The most common pathogen isolated was *Streptococcus pneumoniae* (n = 6), followed by coagulase-negative *Staphylococcus* spp. (n = 5), *Burkholderia cepacia* (n = 4), *Klebsiella pneumoniae* (n = 2), and *Pseudomonas* spp. (n = 2). Of 6 *S. pneumoniae* isolates, 5 were documented within 48 hours of admission, including 2 from children who died. Laboratory parameters at time of admission did not differ significantly between those who survived and those who died, except for hemoglobin (odds ratio [OR] 0.71, 95% confidence interval [CI] 0.59–0.87, p = 0.001), arterial bicarbonate (OR 0.81, 95% CI 0.72–0.91, p<0.001), and serum albumin (OR 0.90, 95% CI 0.87–0.94, p<0.001). Among 1,049 children for whom chest radiographs were taken at time of admission, infiltrates were noted for 643 (61.3%), consolidation for 254 (24.2%), and pleural effusion for 4 (0.4%). No radiographic abnormalities were detected for 173 (16.5%) children. Antiviral drugs had been given to 78 (5.7%) children before admission and to 1,306 (95.6%) children after admission. The most common antiviral drug used was oseltamivir, followed by zanamivir (received by 2 [0.2%] children). An antiviral drug was given within 48 hours of onset of signs for 388 (28.5%) children, including 382 (29.1%) who survived and 6 (11.7%) who died. An antiviral drug was given >48 hours after onset of signs for 783 (57.5%) children, including 748 (57.1%) who survived and 35 (68.6%) who died. Administration of an antiviral drug within 48 hours of onset of signs was associated with a lower risk for death (OR 0.4, 95% CI 0.2–0.9; p = 0.02). Median duration of oseltamivir administration was 5 days (IQR 4–7 days). Among 1,306 children for whom data were available, 982 (75.2%) also received antibacterial drugs.

Among the same 1,306 children for whom data were available, 461 (35.3%) had a concurrent illness (Table 2); 416 (31.9%) had 1 concurrent illness and 65 (4.9%) had 2. Presence of >1 concurrent conditions was associated with a 4-fold increased risk for death (OR 4.4, 95% CI 2.4–8.1; p<0.001). Risk for death was higher for those with chronic lung disease (OR 2.5, 95% CI 1.1–5.6; p<0.02) than for those with other concurrent conditions. Among the 64 (4.7%) children who required inotropic support, 23 (34.3%) survived. Among all 1,362 hospitalized children, 51 (3.7%) died, including 46 (90.2%) in intensive care units and 25 (49%) who were <2 years of age (OR 1.51, 95% CI 0.86–2.64), p = 0.15). Among the 101 children who required mechanical ventilation, 49 (48.5%) died. Among the 1,352 children for whom follow-up data were available, 1,285 (95%) recovered fully and had no sequelae at time of hospital discharge, and 12 (0.9%) recovered but had sequalae (5 pulmonary, 4 neurologic, 2 renal, and 1 pulmonary and neurologic). The mortality rate for children who were <12, <5, and <2 years of age during June–July 2009 was 0.1 death per 100,000 children. Corresponding rates for August 2009–February 2010 were 0.6, 0.9, and 1.3 per 100,000 children, respectively.

**Table 2:** Concurrent conditions in children hospitalized for pandemic (H1N1) 2009, Malaysia, June 18, 2009–March 1, 2010*

Condition	No. (%) children			OR (95% CI)	p value
	Total	Survived	Died		
None	860(63.1)	845 (64.7)	15 (29.4)	0.2 (0.1–0.4)	<0.001
Chronic lung disease	258 (18.9)	246 (18.8)	12 (23.5)	2.5 (1.1–5.6)	0.02
Neuromuscular disease	33 (2.4)	27 (2.1)	6 (11.8)	2.5 (4.5–34.8)	<0.001
Cardiovascular disease	54 (4.0)	46 (3.5)	8 (15.7)	9.8 (3.9–24.3)	<0.001
Renal disease	18 (1.3)	16 (1.2)	2 (3.5)	–	–
Immunosuppression	18 (1.3)	15 (1.1)	3 (5.9)	–	–
Obesity	14(1.0)	13 (1.0)	1 (2.0)	–	–
Malnutrition	14 (1.0)	13 (1.0)	1 (2.0)	–	–

*OR, odds ratio; CI, confidence interval; –, numbers too small to infer from study sample.

## Conclusions

In the tropics, pandemic (H1N1) 2009 is a relatively mild illness in children who have no concurrent condition. Serious complications such as shock and acute respiratory distress syndrome were relatively rare. However, among the small proportion for whom disease was severe, progression was rapid and death occurred within a short period. The case-fatality rate for the hospitalized cohort reported here was 3.7%, comparable to the rates of 0.1%–5.1% documented by others (*4**,**9**,**14*). Hospitalization and mortality rates were proportionally higher for children <2 years of age than for children in other age groups. Severe disease leading to death was more likely for patients who had >1 concurrent condition. Other studies have demonstrated similar findings (*3**,**4**,**7**,**11*).

Data on concurrent conditions can help identify and prioritize patients who need prompt antiviral drug therapy and vaccination in countries with limited resources. Our finding that early administration of an antiviral drug was associated with a lower risk for death concurs with findings of other studies (*3*). Study limitations include the facts that patients with mild cases may not seek (or be brought for) medical attention and that not all cases of ILI were laboratory confirmed as pandemic (H1N1) 2009. Early initiation of antiviral therapy, especially for children with concurrent conditions, may improve outcomes.
